# Photodynamic diagnostic ureteroscopy using the VISERA ELITE video system for diagnosis of upper-urinary tract urothelial carcinoma: a prospective cohort pilot study

**DOI:** 10.1186/s12894-021-00819-2

**Published:** 2021-03-25

**Authors:** Koichiro Wada, Motoo Araki, Ryuta Tanimoto, Takuya Sadahira, Shogo Watari, Yuki Maruyama, Yosuke Mitsui, Hirochika Nakajima, Herik Acosta, Satoshi Katayama, Takehiro Iwata, Shingo Nishimura, Atsushi Takamoto, Tomoko Sako, Kohei Edamura, Yasuyuki Kobayashi, Masami Watanabe, Toyohiko Watanabe, Yasutomo Nasu

**Affiliations:** 1 Department of Urology, Okayama University Graduate School of Medicine, Dentistry and Pharmaceutical Sciences, 2-5-1, Shikata-cho, Kita-ku, Okayama, 700-8558 Japan; 2 Department of Urology, Kagawa Prefectural Central Hospital, 1-2-1, Asahi-machi, Takamatsu, 760-8557 Japan; 3 Department of Urology, Fukuyama City Hospital, 5-23-1, Zao-cho, Fukuyama, 721-8511 Japan; 4 Okayama Urological Research Group: OURG, 2-2-7-1, Ima-cho, Kita-ku, Okayama, 700-0975 Japan

**Keywords:** Photodynamic diagnosis, 5-Aminolevulinic acid, ALA-PDD, Upper urinary tract urothelial carcinoma, VISERA ELITE video system

## Abstract

**Background:**

The advantages of photodynamic diagnostic technology using 5-aminolevulinic acid (ALA-PDD) have been established. The aim of this prospective cohort study was to evaluate the usefulness of ALA-PDD to diagnose upper tract urothelial carcinoma (UT-UC) using the Olympus VISERA ELITE video system.

**Methods:**

We carried out a prospective, interventional, non-randomized, non-contrast and open label cohort pilot study that involved patients who underwent ureterorenoscopy (URS) to detect UT-UC. 5-aminolevulinic acid hydrochloride was orally administered before URS. The observational results and pathological diagnosis with ALA-PDD and traditional white light methods were compared, and the proportion of positive subjects and specimens were calculated.

**Results:**

A total of 20 patients were enrolled and one patient who had multiple bladder tumors did not undergo URS. Fifteen of 19 patients were pathologically diagnosed with UT-UC and of these 11 (73.3%) were ALA-PDD positive. Fourteen of 19 patients were ALA-PDD positive and of these 11 were pathologically diagnosed with UC. For the 92 biopsy specimens that were malignant or benign, the sensitivity for both traditional white light observation and ALA-PDD was the same at 62.5%, whereas the specificities were 73.1% and 67.3%, respectively. Of the 38 specimens that were randomly biopsied without any abnormality under examination by both white light and ALA-PDD, 11 specimens (28.9%) from 5 patients were diagnosed with high grade UC. In contrast, four specimens from 4 patients, which were negative in traditional white light observation but positive in ALA-PDD, were diagnosed with carcinoma in situ (CIS).

**Conclusions:**

Our results suggest that ALA-PDD using VISERA ELITE is not sufficiently applicable for UT-UC. Nevertheless, it might be better particularly for CIS than white light and superior results would be obtained using VISERA ELITE II video system.

*Trial registration*: The present clinical study was approved by the Okayama University Institutional Review Board prior to study initiation (Application no.: RIN 1803–002) and was registered with the UMIN Clinical Trials Registry (UMIN-CTR), Japan (Accession no.: UMIN000031205).

## Background

Photodynamic diagnostic (PDD) technology using 5-aminolevulinic acid (ALA-PDD) is currently being used for the diagnosis of brain tumors in Japan. For patients with bladder cancer, the sensitivity and specificity of ALA-PDD using scopes and video systems produced by Karl Storz SE & Co. KG (Tuttlingen, Germany) can be as high as 75.8% and 68.2%, respectively [1]. Thus, in 2017 Japanese national health insurance approved ALA-PDD for visualization of non-muscle-invasive bladder cancer at the time of transurethral resection of bladder tumors (TURBT). ALA is an amino acid that is naturally present in living animals and plants, as well as in commonly consumed foods and beverages, and protoporphyrin IX (PpIX), which is also present in the human body. Neither compound shows toxicity and the safety of both has been confirmed in healthy individuals [2]. The premise for ALA-PDD is that orally ingested ALA accumulates as PpIX in tumor cells to a greater degree than in normal cells. Upon excitation with visible blue light (375–445 nm), PpIX emits red light (600–740 nm).

Upper tract urothelial carcinoma (UT-UC) includes renal pelvic and ureteral cancer, which are the same as bladder cancer from a pathological perspective. Although standard treatment for UT-UC is total nephroureterectomy and partial cystectomy, techniques involving endoscopic diagnosis and resection with URS have been developed [3, 4]. However, small satellite tumors, flat tumors and carcinoma in situ (CIS) in the upper urinary tract are difficult to diagnose and treat endoscopically [5, 6]. ALA-PDD allows visualization of lesions that cannot be diagnosed by conventional observation using white light, thus increasing the likelihood of accurate diagnosis and effective treatment in patients with UT-UC [7, 8]. ALA-PDD performed for UT-UC patients using a ureteroscope and a video system produced by Karl Storz SE & Co. KG. has been described [9, 10], but no similar study has been carried out using a video system manufactured by Olympus Co., Ltd. (Tokyo, Japan).

The aim of this prospective cohort study was to evaluate the usefulness of ALA-PDD for diagnosis of UT-UC using the VISERA ELITE video system produced by Olympus Co., Ltd.

## Methods

All methods were carried out in accordance with relevant guidelines and regulations.

### Study design

We carried out a pilot, prospective interventional, non-randomized, non-contrast and open label cohort study. Enrollment began following approval by the review board. Patients who were suspected of having UT-UC and were undergoing ureterorenoscopy (URS) were enrolled. Since this study was a pilot study, 20 cases were considered based on the annual number of cases treated at our institution. The primary endpoint was the proportion of positive subjects and secondary endpoints were sensitivity, specificity, positive predictive value (PPV), negative predictive value (NPV) and adverse events for ALA-PDD.

### Patients

Patients that were enrolled in the study were adults who were suspected of having UT-UC and were undergoing URS. All patients were aware of the conditions associated with the disease, and provided informed consent to participate. All enrolled patients agreed to use contraception until 1 month after the administration of 5-ALA. Exclusion criteria were patients with porphyria, allergy to porphyrin or 5-ALA, hepatic dysfunction, use of drugs that induced photosensitivity, pregnancy, or severe underlying diseases such as cardiovascular diseases or infection.

### Protocols

On the day of the URS, 20 mg/kg of 5-aminolevulinic acid hydrochloride (5-ALA HCl) was orally administered over 180 min (range: 120–240 min) before the scheduled insertion time for the ureteroscope. After oral administration of 5-ALA HCl, patients drank 250 mL of an L-arginine-intensive drink and 500 mL of fruit juice to maintain blood pressure. At the time of examination, normal observations using white light and ALA-PDD using visible blue light were carried out using a two-color LED light source (ALADUCK LS-DLED, SBI Pharmaceuticals, Tokyo, Japan). All endoscopic images were recorded.

After the procedures, patients had proper shading to prevent photosensitivity for up to 48 h after ingesting 5-ALA HCl and light exposure of less than 500 lx was maintained. Hematologic and biochemical examinations were performed on postoperative days (POD) 1 and 3 and the occurrence of adverse events was noted.

### 5-ALA (5-aminolevulinic acid)

5-ALA HCl was supplied as a white powder similar to that for ALAGLIO by SBI Pharmaceuticals (SBI Pharmaceuticals, Tokyo, Japan). The chemical compound was stored according to strict quality control guidelines and detailed usage records were maintained by the Division of Clinical Research of New Drugs and Therapeutics in the Center for Innovative Clinical Medicine, Okayama University Hospital.

### Endourological techniques

The endourological techniques and URS procedures performed at our institution for observation of the upper urinary tract and ablation of tumors were previously described [11, 12]. All the patients underwent URS using “no-touch” technique, which is without any insertion of guidewire or ureteral catheter prior to ureteroscope, under general anesthesia in a lithotomy position. Before the procedures, 10 μM PpIX in a glass vial was observed using blue light and the ureteroscopes were confirmed to glow red. After cystoscopy, a 6/7.5 Fr semi-rigid ureteroscope (E-line, Richard Wolf, Knittlingen, Germany) was inserted with or without a guidewire as high as possible, and then changed to an 8.4 or 7.95 Fr flexible ureteroscope (URF-P5 or P6, Olympus Co. Ltd., Tokyo, Japan). At observation, white light and blue light, switched with a foot pedal, were alternately used. When abnormalities, such as a tumor or red fluorescence, were observed, cold cup biopsies were performed using 3.3 Fr Forceps™ (Cook Medical, Bloomington, IN, USA) without any hemostatic technique. Among patients who had preoperative positive urine cytology and had no abnormality, cold cup biopsies from the renal pelvis and ureter were randomly performed. All endourological procedures were performed using the VISERA ELITE video system and camera head produced by Olympus Co., Ltd.

### Data analysis

The observational results and pathological diagnoses, the proportion of positive subjects, positive specimens, sensitivity, and specificity were calculated. Adverse events were also investigated and severity was categorized using the Clavien-Dindo classification.

## Results

Characteristics of the 20 patients enrolled in this study are shown in Table 1. A total of 5 patients (25%) had positive preoperative urine cytology and no abnormalities on CT urography. Another 5 patients (25%) had abnormalities on CT urography but negative urine cytology.

**Table 1:** Patient characteristics before URS using ALA-PDD

	Number of patients	(%)
Median age (IQR)	71	(67–79)
Gender		
Male	16	(80)
Female	4	(20)
Co-morbidities		
Malignancy	2	(10)
Urothelial carcinoma	1	(5)
UT-UC	0	
Bladder cancer	1	(5)
Other	0	
Cardiovascular diseases	5	(25)
Diabetes mellitus	6	(30)
Other	4	(20)
None	7	(35)
Laterality		
Right	8	(40)
Left	9	(45)
Bilateral	2	(10)
Unknown	1	(5)
Disease site		
Renal pelvis	6	(30)
Clinical stage		
< cT2	3	(15)
≥ cT2	3	(15)
Ureter	7	(35)
Clinical stage		
< cT2	4	(20)
≥ cT2	3	(15)
Unknown (no lesion on CT or MRI)	7	(35)
Urine cytology		
Positive	13	(65)
Negative	7	(35)

**Fig. 1:**
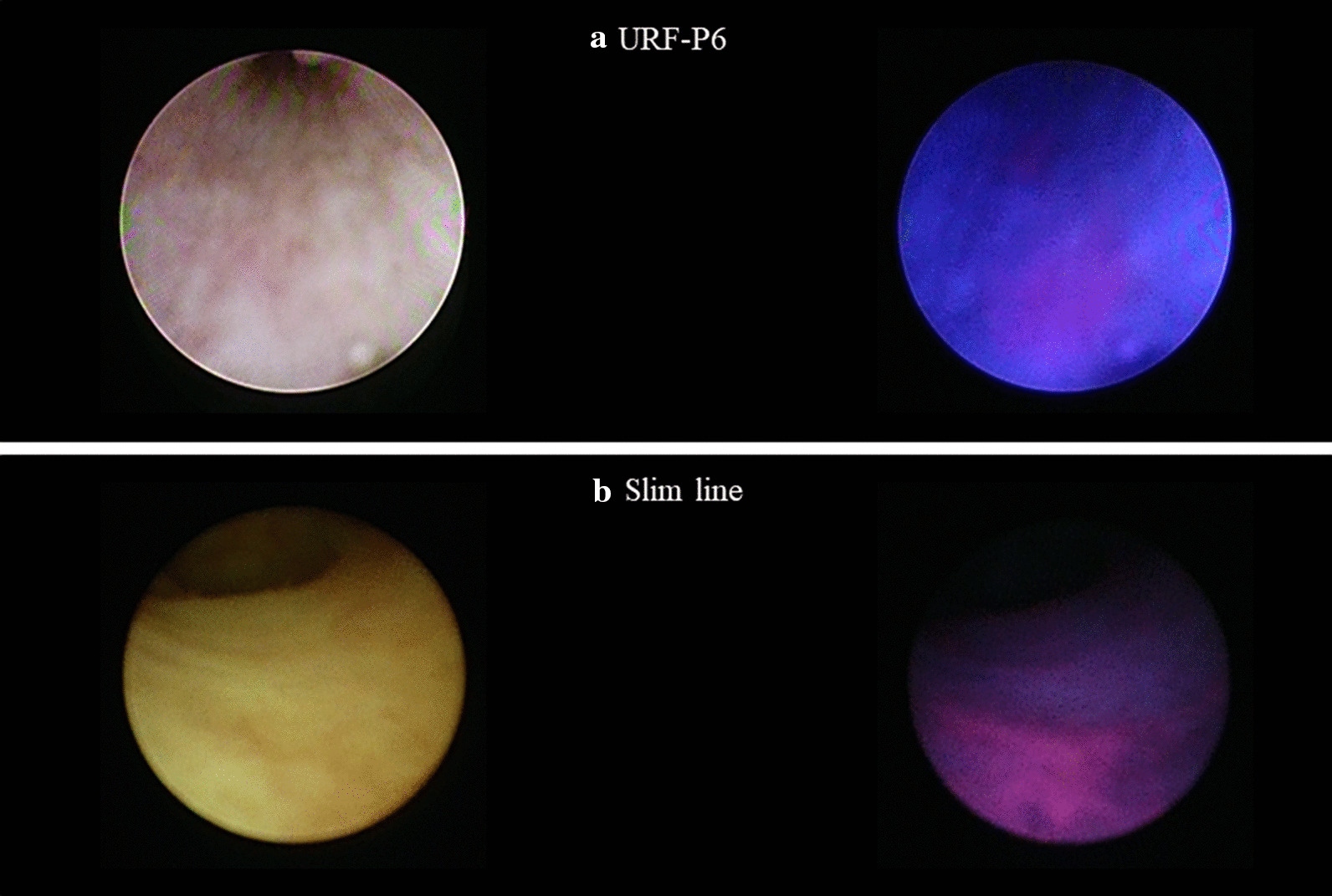
Two different white light negative and ALA-PDD positive lesions using flexible URS (URS-P6, **a**) and semi-rigid ureteroscopy (Slim line, **b**) followed by diagnosis with carcinoma in situ

The median (IQR) time from oral administration of 5-ALA HCl to biopsy was 246 (227–269) minutes. One patient had multiple tumors in the bladder and URS was aborted. Among the 19 remaining patients, biopsy was not performed because of absence of any abnormality in one patient, for whom URS was performed as follow-up. Fifteen patients were pathologically diagnosed with UT-UC and of these, 11 (73.3%) were ALA-PDD positive. Fourteen of 19 patients were ALA-PDD positive and of these, 11 were pathologically diagnosed with UC. Among the 50 specimens that are ALA-PDD positive, 25 (50%) were pathologically diagnosed with UC, 17 (34%) were not malignant, and the status for 8 (16%) could not be determined because the specimens were too small. Among the 7 patients that were ALA-PDD positive and white light negative (Fig. 1), 4 were diagnosed with CIS. Of the 38 specimens from 12 patients that showed no abnormalities under examination by both white light and ALA-PDD, 11 specimens (28.9%) from 5 patients were diagnosed with high grade UC. Comparing white light and ALA-PDD, 4 specimens taken from definite tumors in 2 patients were ALA-PDD negative but diagnosed with UC. On the other hand, 4 specimens which were white light negative and ALA-PDD positive taken from 4 patients were diagnosed with CIS. Of the 31 specimens diagnosed with high grade UC, 18 and 13 (58% and 42%) were ALA-PDD positive and negative, respectively. For specimens from patients with low grade UC, 7 (78%) of 9 were ALA-PDD positive, but 2 (22%) specimens were ALA-PDD negative.

For observational accuracy calculations, 2 patients who had pathological diagnoses were excluded and 18 patients were enrolled. Based on the results for these 18 patients, the sensitivity and specificity of ALA-PDD were calculated to be 80.0% and 33.3%, respectively, whereas for white light observation the respective values were 86.7% and 66.7% (Table 2). Considering biopsy specimens that could be diagnosed as malignant or benign (n = 92), the sensitivity of white light observation and ALA-PDD was the same at 62.5%, whereas the specificities were 73.1% for white light and 67.3% for ALA-PDD, respectively. The PPV and NPV of ALA-PDD were 59.5% and 70.0%, respectively. The combined observation had the highest sensitivity at 72.5%, but the lowest specificity at 51.9%.

**Table 2:** Diagnostic accuracy of normal white light observation, ALA-PDD and combined observation

	White light			ALA-PDD			Combined observation		
	Positive	Negative	Total	Positive	Negative	Total	Positive	Negative	Total
*A. Analysis by participant (n* = *18)*									
Pathological diagnosis									
UC	13	2	15	12	3	15	15	0	15
Non-UC	1	2	3	2	1	3	2	1	3
Total	14	4	18	14	4	18	17	1	18
Accuracy of each observational method	Sensitivity		86.7%	Sensitivity		80.0%	Sensitivity		100.0%
	Specificity		66.7%	Specificity		33.3%	Specificity		33.3%
	PLR^a^		2.6	PLR		1.2	PLR		1.5
	NLR^b^		0.2	NLR		0.6	NLR		-
	FPR^c^		13.3%	FPR		20.0%	FPR		0.0%
	FNR^d^		33.3%	FNR		66.7%	FNR		66.7%
	PPVe		92.9%	PPV		85.7%	PPV		88.2%
	NPV^f^		50.0%	NPV		25.0%	NPV		100.0%

	Normal observation			ALA-PDD			Combined observation		
	Positive	Negative	Total	Positive	Negative	Total	Positive	Negative	Total
*B. Analysis by biopsy specimen (n* = *92)*									
Pathological diagnosis									
Urothelial carcinoma	25	15	40	25	15	40	29	11	40
Non-urothelial carcinoma	14	38	52	17	35	52	25	27	52
Total	39	53	92	42	50	92	54	38	92
Accuracy of each observational method	Sensitivity		62.5%	Sensitivity		62.5%	Sensitivity		72.5%
	Specificity		73.1%	Specificity		67.3%	Specificity		51.9%
	PLR		2.3	PLR		1.9	PLR		1.5
	NLR		0.5	NLR		0.4	NLR		0.5
	FPR		37.5%	FPR		37.5%	FPR		27.5%
	FNR		26.9%	FNR		32.7%	FNR		48.1%
	PPV		64.1%	PPV		59.5%	PPV		53.7%
	NPV		71.7%	NPV		70.0%	NPV		71.1%

^a^PLR: positive likelihood ratio

^b^NLR: negative likelihood ratio

^c^FPR: false positive ratio

^d^FNR: false negative ratio

^e^PPV: positive predictive value

^f^NPV: negative predictive value

For adverse events, all patients took the first 50 mL of drug solution including 5-ALA HCl; however, two patients complained of nausea and one vomited 2 h after taking 5-ALA HCl. Other symptoms included elevated serum aminotransferase that required no additional treatment (Clavien-Dindo Grade I) in 6 patients and hypotension requiring vasopressors (Grade II) in one patient. No allergic episodes, cardiovascular complications or photosensitivity were observed in our study.

## Discussion

In this prospective cohort study to evaluate the effectiveness of ALA-PDD for UT-UC diagnosis using the VISERA ELITE video system, the sensitivity and specificity were 62.5% and 67.3%, respectively. Of the 38 specimens that were randomly biopsied without any abnormality under examination by both white light and ALA-PDD, 11 specimens (28.9%) from 5 patients were diagnosed with high grade UC. On the other hand, four biopsy specimens that had abnormal findings with ALA-PDD but not with normal white light observation were diagnosed with CIS.

**Fig. 2:**
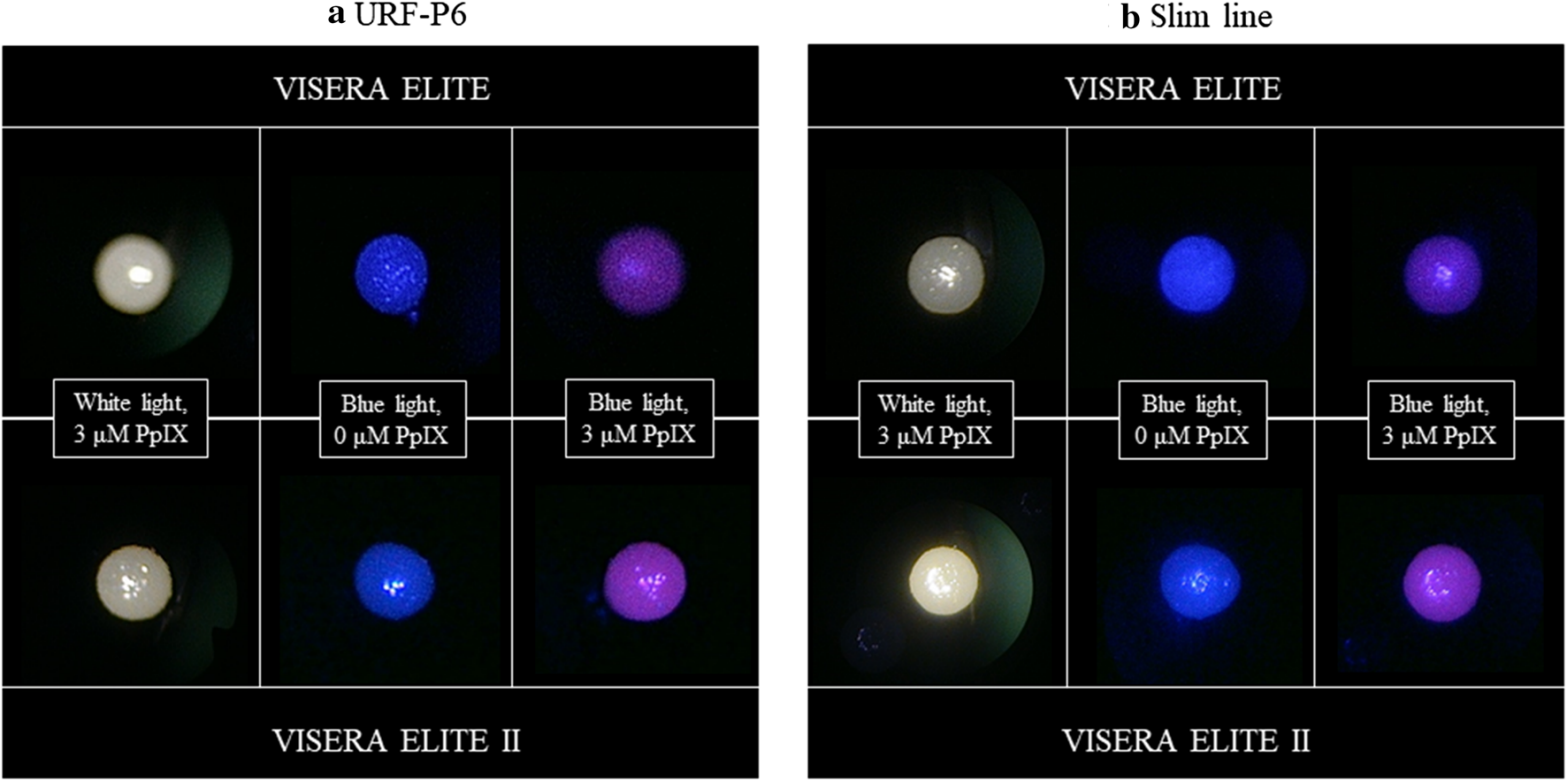
Ex vivo experiments for evaluation of ELITE and ELITE II. Cotton swabs containing 0 or 3 μM PpIX are observed using the same flexible URS (URS-P6, **a**) and semi-rigid ureteroscopy (Slim line, **b**) by white light or blue light (ALA-PDD) at a distance of 5 mm. In both case of flexible URS and semi-rigid ureteroscopy, a higher fluorescent intensity with ELITE II (white arrows) compared to ELITE (black arrows) is seen observing the cotton swabs containing 3 μM PpIX by blue light

ALA-PDD was approved only for bladder cancer by Japanese national health insurance in 2017. This approach is expected to have value not only for bladder cancer diagnosis but also for treatment, namely complete resection at TURBT [1, 13–16]. On the other hand, studies describing photodynamic treatment using ALA (ALA-PDT) [17] and some pilot studies [18, 19] involving ALA-PDD for UT-UC reported that ALA-PDT and ALA-PDD could be effective and useful even for UT-UC (Table 3). Among studies concerning ALA-PDT and -PDD conducted since 2012, most used ureteroscopes with a video system produced by Karl Storz SE & Co. KG (Tuttlingen, Germany) to diagnose ALA-PDD and this technique was shown to have higher accuracy for UT-UC diagnosis than methods involving white light [10, 20–22] and CT urography [22]. Thus, before carrying out the present study, we confirmed that PpIX fluorescence could be observed using a ureteroscope with the VISERA ELITE video system [23]. Our present study revealed that sensitivity and specificity values for ALA-PDD for UT-UC were lower than other studies for UT-UC and slightly lower than those for bladder cancer. These outcomes could be due to differences in: (1) cystoscopes and ureteroscopes; (2) image quality; and (3) video system quality. First, images obtained using ureteroscopy are dark and small because ureteroscopes are longer and thinner than cystoscopes. Second, image processing software that optimizes gradations of collected images has not been available yet. Third, neither the VISERA ELITE nor camera head are designated for ALA-PDD, and thus surgeons must insert a special filter between the camera head and the endoscope. Use of the newest video system such as the VISERA ELITE II could produce a higher sensitivity and specificity since we did perform additional ex vivo experiments to evaluate which video system was better for ALA-PDD using ureteroscopy, and found that images produced by the VISERA ELITE II were superior to those obtained using VISERA ELITE (Fig. 2).

**Table 3:** Previous studies describing use of ALA-PDT/PDD to diagnose UT-UC

References	Country	Study design	Objective	Sample size	Video system supplier	Sensitivity (%)	Specificity (%)
Waidelich [17]	Germany	Pilot study	PDT^a^	4	Not stated	–	–
Somani et al. [18]	UK	Pilot study	PDD^b^	4	Karl Storz	–	–
Audenet et al. [19]	France	Review	–	–	–	–	–
Ahmad et al. [20]	UK	Prospective cohort	PDD	26	Karl Storz	100	62.5
Aboumarzouk et al. [21]	UK	Prospective cohort	PDD	32	Karl Storz	96	100
Aboumarzouk et al. [22]	UK	Prospective cohort	PDD	30	Karl Storz	94	100
Bondad et al. [25]	UK	Retrospective	Unknown	24	–	–	–
Kata et al. [9]	UK	Review	PDD	–	Karl Storz	–	–
Kata et al. [10]	UK	Prospective cohort	PDD	54	Karl Storz	95.8	96.6
Osman et al. [7]	Bahrain	Review	–	–	–	–	–
This study (2019)	Japan	Prospective cohort	PDD	20	VISERA ELITE, Olympus	62.5	67.3%

^a^*PDT* photodynamic therapy

^b^*PDD* photodynamic diagnosis

Interestingly, 11 of the 38 (28.9%) specimens that exhibited no abnormalities in either white light or ALA-PDD examinations were pathologically diagnosed with high grade UC. The reasons for this result are unclear, but some abovementioned factors may have contributed to this outcome. To our knowledge there are no studies that examined random biopsy samples taken from the upper urinary tract, and additional investigations are needed.

According to European guidelines [24], diagnostic ureteroscopy is strongly recommended to diagnose UT-UC tumor type and tumor grade. Furthermore, endoscopic management for UT-UC is the primary treatment option as a kidney-sparing surgery for patients with low-risk tumors (unifocal tumor; tumor size < 2 cm; low-grade cytology; low-grade URS biopsy; no invasive aspect on CT urography). Although in our study 2 specimens from one patient with low grade UC were ALA-PDD negative, low grade UC can be positive even in normal white light observation. In addition, 4 specimens diagnosed with CIS were negative in normal observation but positive in ALA-PDD. Hence, our results indicate that ALA-PDD might be an effective method for detection of CIS in the upper urinary tract.

Adverse events of ALA-PDD have been reported as liver toxicity, allergic episode, cardiovascular complications, photosensitivity, hypotension, nausea and vomiting [9, 25]. In our study, no severe adverse events were observed outside of hypotension in one patient because of some preventative measures such as oral intake before the procedure, shading for 48 h to avoid photosensitivity, and other factors. However, one patient who had vomited 2 h after administration of 5-ALA was diagnosed with high grade UC while any abnormality could not be observed under examination by both white light and PDD. The fact suggests the possibility that vomiting had some effect on ALA-PDD.

The present study has several limitations. Although this pilot study was prospective, it was performed at a single center, was non-randomized and included small number of patients (n = 20). Second, there were 2 patients who underwent protocol URS after endoscopic laser ablation and cognitive bias could be associated with the previous procedure. Third, we evaluated the usefulness of ALA-PDD only in the diagnostic process and not the treatment process. Fourth, we did not compare endoscopic devices including semirigid/flexible ureteroscopes, light cables, and video systems, and did not evaluate their compatibility with ALA-PDD. Despite these limitation, the ureteroscopy with ALA-PDD might be more useful for diagnosis in patients with UT-UC, particularly with CIS.

## Conclusions

Our results of this pilot study suggest that ALA-PDD using VISERA ELITE is not sufficiently applicable for UT-UC. Nevertheless, it might be better particularly for CIS than white light and superior results would be obtained using VISERA ELITE II video system. Further studies on patient selection, endoscopic equipment and image processing methods are needed for the broad application of ALA-PDD for UT-UC diagnosis.

## Data Availability

The full dataset collected and analyzed during this study include individual data. Thus, it is available only from the corresponding author on reasonable request.
